# Primary scrotal lipoma in a child: a rare case report and review of literature

**DOI:** 10.3389/fped.2024.1360943

**Published:** 2024-10-30

**Authors:** Meng Kong, Yuexia Bai, Jinhua Jia, Chuanyang Liu, Shisong Zhang

**Affiliations:** ^1^ Department of Pediatric Surgery, Children’s Hospital Affiliated to Shandong University, Jinan, China; ^2^ Department of Pediatric Surgery, Jinan Children’s Hospital, Jinan, China; ^3^ Department of Pathology, Children’s Hospital Affiliated to Shandong University, Jinan, China

**Keywords:** scrotal lipoma, child, ultrasonography, resection, pathological

## Abstract

Lipoma is a common benign tumor of the body surface, while scrotal lipoma is relatively rare. We report a case of scrotal lipoma in a 5-year-old boy, which presented as a progressively enlarging scrotal mass. Scrotal ultrasonography highly suggested a lipoma, and a scrotal mass resection was performed, leading to a final pathological diagnosis of benign scrotal lipoma.

## 1 Introduction

Lipoma is a common benign tumor of the body surface (1), with a prevalence rate of 2.1 per 1,000 people (2). It grows slowly and is more likely to occur in the neck, shoulder, back, and thigh, while it is less common in the genitourinary system, and even rarer in the scrotum. So far, only a few cases of scrotal lipomas have been reported in the literature, the incidence of giant lipomas is only 1% of lipomas (3, 4). The clinical presentation of scrotal lipoma is non-specific, with most patients having no obvious symptoms and most being diagnosed due to the incidental discovery of scrotal masses causing scrotal discomfort or swelling. Scrotal lipoma can occur at any age, with adults being more commonly affected (4). We report a case of a 5-year-old boy with scrotal lipoma, which grew rapidly within 6 months. Concerned about the possibility of malignant transformation, his parents sought treatment at our hospital, where complete resection of the right scrotal mass was performed. The pathological diagnosis confirmed it as a benign scrotal lipoma.

## 2 Case report

**Figure 1:**
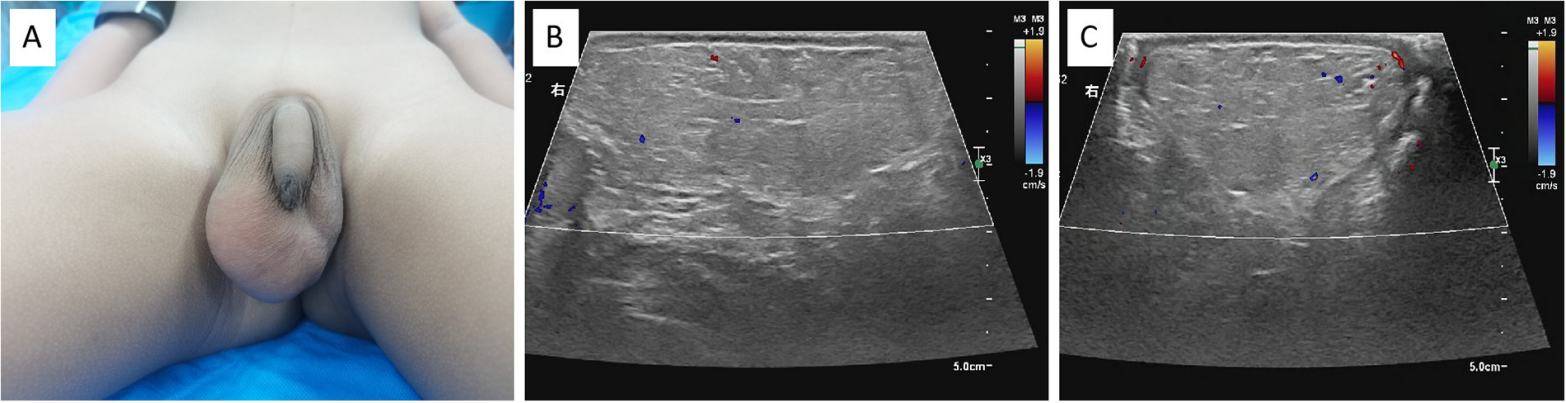
**(A)** A lipoma can be seen in the right scrotum of the child. **(B)** The ultrasound revealed a hypoechoic lipoma in the right scrotum with a clear boundary (transverse section). **(C)** The ultrasound showed the longitudinal section of the scrotal lipoma.

The patient was a 5-year-old male who was admitted to our hospital with “a right scrotal mass that has been present for 6 months.” The parents discovered the mass, about the size of an egg, on the right scrotum of their child 6 months ago, but the child had no symptoms. The parents did not pay much attention due to work commitments. However, over the past 6 months, the mass has noticeably increased in size, leading them to seek evaluation at our hospital. No special treatment was given outside of the hospital, and the patient presented to our hospital for diagnosis and treatment. The patient had no remarkable personal or family history. Physical examination showed no redness or swelling of the scrotal skin, with a skin temperature of the scrotum of 34.7℃ (measured by an infrared thermometer). The scrotum was asymmetric, with the right side significantly larger than the left. A soft mass of approximately 6.0 cm × 4.0 cm × 3.0 cm in size was palpable in the right scrotum, with no pressure or localized tenderness and a smooth surface. The results of the transillumination test were negative, and compressibility was negative. There was no sign of inguinal hernia. Normal-sized testicles were palpable on both sides of the scrotum. There was no palpable lymph node in the abdominal and inguinal examination (Figure 1A). Laboratory examinations showed no abnormality. The diagnostic results of a complete blood count, tumor markers such as human chorionic gonadotropin (HCG) and lactate dehydrogenase (LDH), and chest x-ray were within normal limits. Scrotal ultrasonography results showed a low homogenous solid mass measuring approximately 5.8 cm × 3.9 cm × 2.4 cm located below the right testis in the scrotal wall, with clear boundaries, heterogeneous internal echo, and echoes similar to adipose tissue. No obvious hernia sac echo or hypoechoic area was detected in the bilateral inguinal region. The size and echogenicity of both testicles were normal, with the right testicle measuring approximately 1.4 cm × 0.8 cm and the left testicle measuring approximately 1.5 cm × 0.8 cm. Color Doppler flow imaging showed sparse punctate blood flow signals within the mass. The results suggested a scrotal lipoma inside the right scrotal wall (Figures 1B,C).

**Figure 2:**
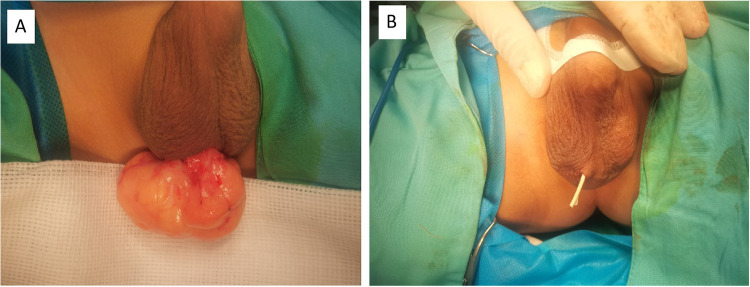
**(A)** A significant lipoma was noted in the right scrotum during the operation. **(B)** The appearance of the scrotum after surgery, and a rubber drainage strip was placed in the incision.

The patient underwent right scrotal mass excision surgery under general anesthesia. The surgical procedure was as follows: a transverse incision of approximately 2.0 cm was made in the middle of the right scrotum, and the skin and subcutaneous fat were dissected layer by layer. A large yellow fatty tissue measuring approximately 6 cm × 5 cm × 4 cm was found in the scrotum, with a relatively intact capsule, clear boundaries, lobulated appearance, and fascial septa. Some of the fatty tissue was connected to the spermatic cord and was completely detached (Figure 2A). Then, the testicle was fixed in the scrotal skin and fascia, and no residual mass was found during the operation. A rubber drainage tube was placed after the incision was closed (Figure 2B). The tumor was completely resected and sent for pathological examination. After the operation, the patient was safely transferred back to the ward and given hemostatic and symptomatic treatments. The drainage tube was removed on the first day after surgery, and the patient was discharged smoothly on the third day.

**Figure 3:**
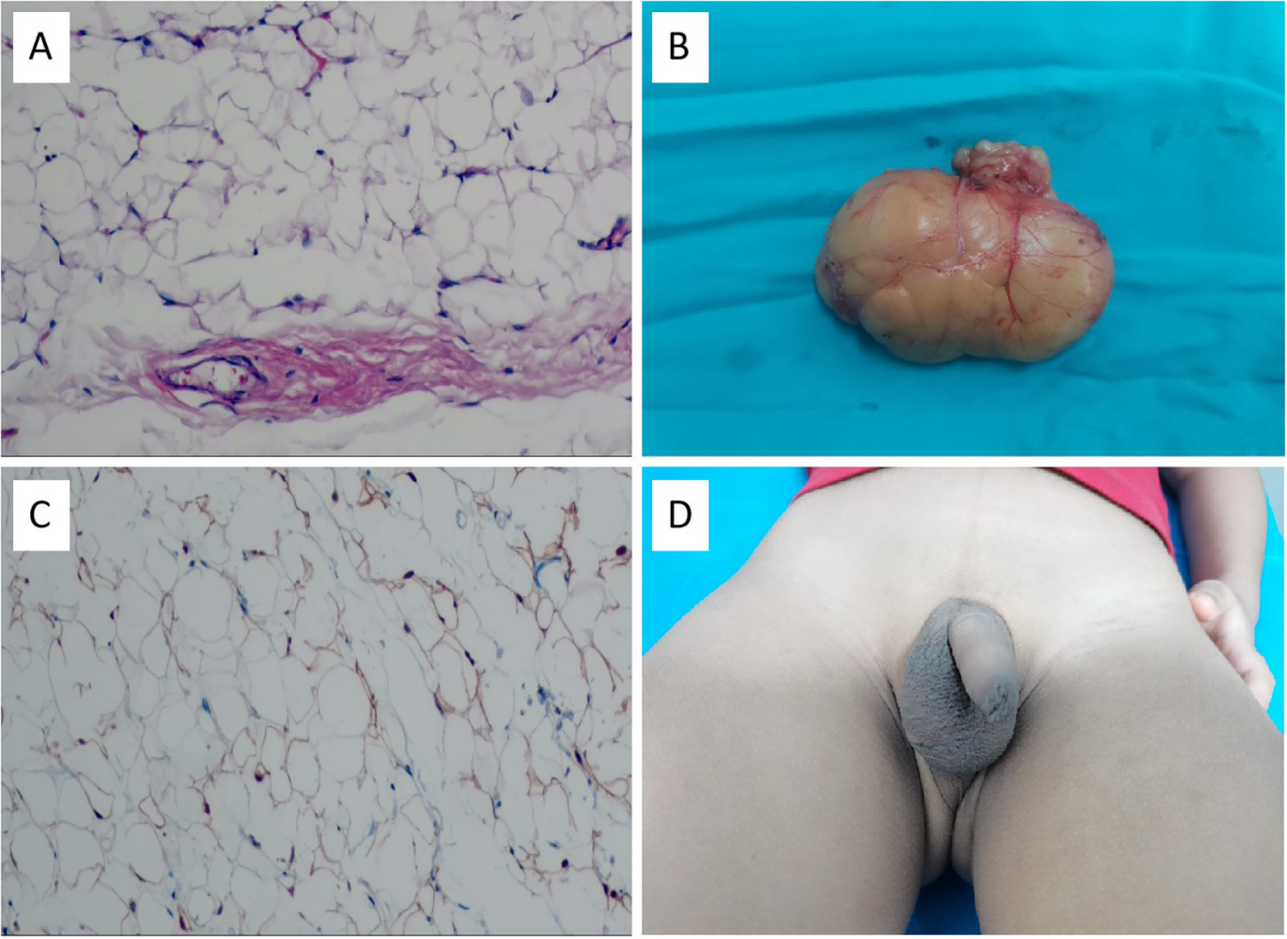
**(A)** Appearance of the excised scrotal lipoma. **(B)** Hematoxylin and eosin staining showed that tumor tissues were composed of mature fat cells and rich in lipid droplets. The cell nucleus is displaced to the periphery. (Hematoxylin and eosin staining,  × 100). **(C)** Immunohistochemical staining revealed positive S100 expression within the nuclei of adipocytes. (Immunohistochemical staining,  × 100). **(D)** Scrotal appearance 6 months after the surgery.

**Figure 4:**
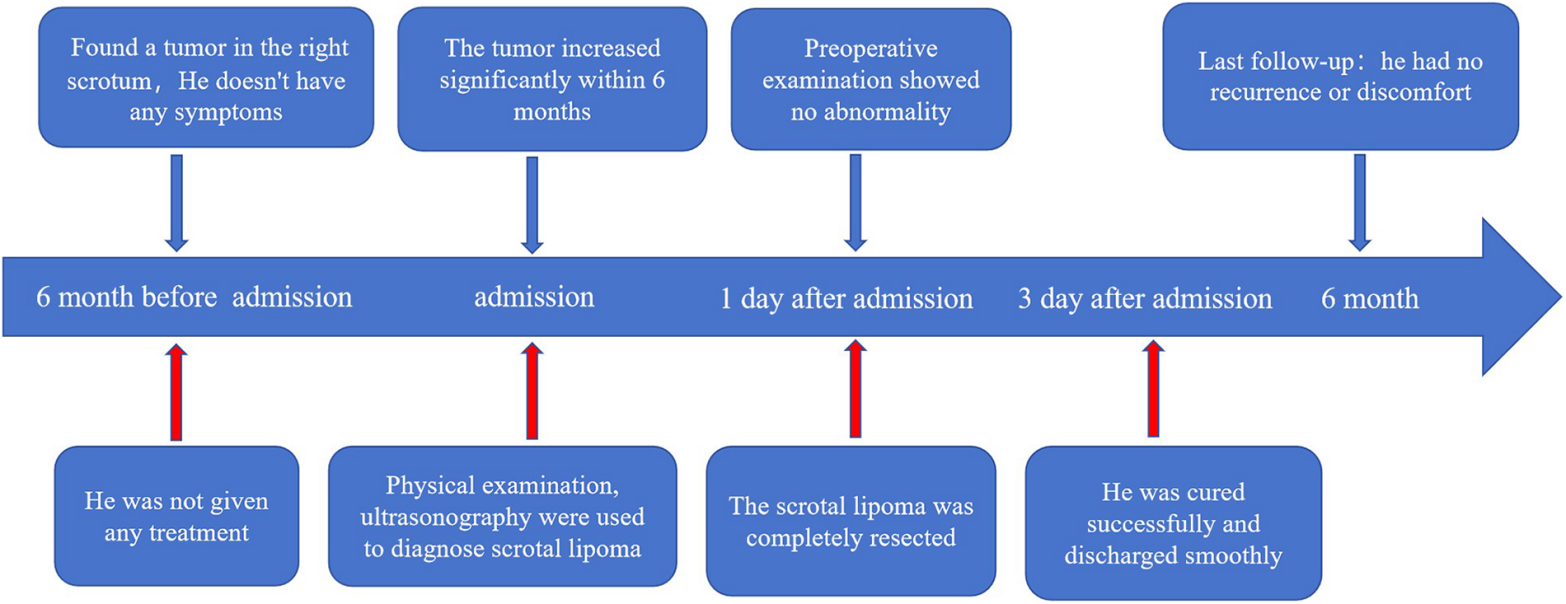
Complete timeline, including diagnosis, surgery, postoperative recovery, and follow-up.

The pathological findings showed typical components of scrotal lipoma (Figure 3A). The postoperative pathology report showed that the resected mass was 6.0 cm × 3.5 cm × 2.9 cm in size, with a smooth surface and intact capsule. The cut surface was grayish yellow and soft. Electron microscopic examination revealed that the tumor tissue was composed of mature adipocytes, which were round or polygonal in shape and contained abundant lipid droplets. The tumor cells were separated into lobules by thin fibrous septa, and the diagnosis was right scrotal lipoma (Figure 3B). Immunohistochemical staining revealed positive S100 expression within the nuclei of adipocytes (Figure 3C). The patient had no recurrence or discomfort during the 6-month follow-up period (Figure 3D, Figure 4).

## 3 Discussion

Scrotal lipoma is a rare benign tumor of mesenchymal origin, and its etiology and pathogenesis are not yet clear (5). Literature reports suggest that scrotal lipoma may be a congenital disease (6), and it may also be due to the development of local pluripotent cells into adipocytes (7). In addition, studies have found that scrotal lipoma may be related to benign symmetric lipomatosis and mammary development (8–10). Our case report describes a 5-year-old boy with a rare lipoma of the scrotum. However, upon careful examination, no symmetric fatty masses or breast development were found in other parts of the patient's body. Fat tumors originating from mesothelial cells can occur in the spermatic cord, appendix testis, parietal tunica vaginalis, and subcutaneous tissue of the scrotum, and a lipoma originating from the subcutaneous fat cells of the scrotal wall is called a primary scrotal lipoma. According to the different origins and locations of growth, it can be divided into three categories (1): (1) tumors originating from the spermatic cord and growing inside the scrotum; (2) tumors originating from the spermatic cord and growing inside the spermatic cord; and (3) tumors originating and growing within the scrotal wall. Scrotal lipomas are generally solitary, and they are less common in the scrotal wall than in the scrotum itself (1), whereas this patient's lipoma occurred on the right side. Scrotal lipomas have a soft and elastic texture, and they are generally not adhered to the surface skin or surrounding tissues and have good mobility. However, if the disease history is long, it may be adhered to surrounding tissues and even undergo malignant transformation (11).

Scrotal lipomas do not have specific clinical manifestations, and patients often come for medical treatment only when the tumor is accidentally discovered during physical examination or when the tumor continues to enlarge and causes obvious scrotal swelling and pain (8). Although the child has not experienced any discomfort since the onset of the disease, his parents noticed a significant increase in the size of the scrotal tumor over the past 6 months. Concerned about the possibility of malignant transformation, they came to our hospital to seek treatment. Diagnosis of scrotal lipoma before surgery can be relatively difficult and is prone to misdiagnosis. Ultrasound, computed tomography (CT), and magnetic resonance imaging (MRI) can help us diagnose lipoma. The advantages of color Doppler ultrasound examination are its economy, convenience, and practicality, and the most important thing is that it has no ionizing radiation, which can be used as a first auxiliary examination. It usually shows a low-echo solid tumor with an intact capsule and interlaced hypoechoic areas, with clear boundaries and no obvious blood flow signals, but it can show septa (12). Compared with ultrasound, CT examination has a high specificity, and it clearly shows the size of the tumor in the scrotum, which appears as a nodular fatty shadow with a lobulated appearance and clearly shows the relationship between the lipoma and the testis and epididymis (4). Magnetic resonance imaging can be used to determine the diagnosis of lipoma, narrow the diagnostic range, and help accurately evaluate the lipoma. Lipoma has high signal intensity on T1- and T2-weighted images and also has signal intensity loss on frequency-selective fat-saturated images (13).

However, the final diagnosis of scrotal lipoma depends on pathological examination, which is the gold standard for definitive diagnosis. It appears yellow on the cut surface, soft in texture, lobulated in shape, with an intact capsule and clear boundaries. Histopathological examination revealed mature adipocytes in the tumor under the microscope, which were separated by unequal amounts of fibrous connective tissue into irregularly sized fat lobules. The number of blood vessels was relatively small (14). After admission, the patient underwent a transvaginal ultrasound examination of the scrotum, which showed a low-echo lesion located within the scrotal wall below the testicle on the right side. The boundary was clear, the internal echo was heterogeneous, and the echo was consistent with fat tissue. There was a sparse spotty blood flow signal within the lesion. No obvious hernia sac echo or hyperechoic area was identified in both inguinal fossa. The size and echo of both testes were normal. The result suggested a right scrotal lipoma. Unfortunately, we did not perform a radiological examination before the operation. Therefore, the purpose of the operation was to complete the resection of the lesion to prevent possible malignant transformation and to minimize damage to surrounding tissues as much as possible. Fortunately, we found that the tumor originated from the spermatic cord and grew into the scrotum during the operation. We completed the resection of the tumor and performed an orchidectomy to prevent the testicle from twisting. The postoperative pathological diagnosis was a benign lipoma of the scrotum.

In clinical practice, scrotal lipomas in children need to be differentiated from the following diseases (15). (1) Testicular tumors grow relatively rapidly, have a hard texture, unclear boundaries with the testis, and ultrasound and CT suggest solid lesions inside the testis. (2) Inguinal hernia: the intestinal or greater omentum protrudes from the internal ring into the scrotum, and the hernia can generally be reduced when lying down. If the contents are intestinal, bowel sounds may be heard, and the transillumination test is negative. (3) Hydrocele of the tunica vaginalis: a cystic mass can be palpated in the scrotum, and the testis is generally not easy to touch. The transillumination test is generally positive, and ultrasound suggests a cystic hypoechoic area in the scrotum. (4) Lipoblastoma: benign lipoblastoma is a rare solitary tumor, mostly occurring in infants, with 80% occurring before the age of 3, so it is also known as embryonal lipoma. It is a localized, lobulated, or diffuse mass resembling fetal adipose tissue. Tumors are most commonly seen before age 3. It occurs occasionally in newborn or older children and tends to be male. The extremities are most commonly involved, but the mediastinum, retroperitoneum, trunk, head and neck, and various organs (lungs, heart, parotid gland) have also been reported (16). (5) In addition, for lipomas, the possibility of malignant tumors should also be ruled out. Liposarcoma is one of the most common soft tissue sarcomas, accounting for 7%–27% of all soft tissue sarcomas (17, 18). Although the ultrasound showed a low probability of malignant lipoma and the actual histological results showed benign, for the above reasons, we performed a complete excision. Therefore, careful preoperative physical examination and necessary auxiliary examination are very helpful for the diagnosis of the disease.

At present, it is believed that small scrotal lipoma can not be treated, and follow-up observation can be done (19). Surgery is required for tumors that are growing rapidly, for malignant changes that must be eliminated, or for large sizes (diameter > 10 cm or weight > 1 kg) resulting in discomfort such as swelling and pain of the scrotum (20). Although the child, in this case, has no discomfort at present, the parents found that the scrotal mass of the child grew rapidly within 6 months, were very worried about the possibility of malignant changes, and had a strong desire for surgery. Therefore, we decided to perform surgical resection after careful consideration. The surgical resection of lipoma is an effective and reliable treatment method, and the complete resection of the tumor should be ensured during the operation to reduce recurrence. Whether giant asymptomatic lipomas can produce pressure effects similar to testicular torsion, affecting normal testicular blood flow, and whether early and complete resection is necessary due to their potential hazards still require more effective case support and longer-term close follow-up.

## 4 Conclusion

Lipomas of the scrotum are very rare, especially in children, and can be misdiagnosed clinically. If malignant degeneration is suspected before surgery, surgical resection may be the ideal treatment strategy.

## Data Availability

The original contributions presented in the study are included in the article/Supplementary Materials, further inquiries can be directed to the corresponding author.
